# Cephalometric assessment regarding craniocervical posture in orthodontic patients

**DOI:** 10.1038/s41598-022-26243-6

**Published:** 2022-12-16

**Authors:** Vlad Tiberiu Alexa, Aurora Doris Fratila, Camelia Szuhanek, Daniela Jumanca, Dacian Lalescu, Atena Galuscan

**Affiliations:** 1 Orthodontic Research Center (ORTHO-CENTER), Faculty of Dental Medicine, “Victor Babeş” University of Medicine and Pharmacy, Eftimie Murgu Sq. No 2, 300041 Timisoara, Romania; 2 Faculty of Dental Medicine, Ludwig-Maximilian-University Munich, Goethestraße 70, 80336 München, Germany; 3 Faculty of Dental Medicine, “Victor Babeş” University of Medicine and Pharmacy, Eftimie Murgu Sq. No 2, 300041 Timisoara, Romania; 4 Translational and Experimental Clinical Research Center in Oral Health (TEXC-OH), 14A Tudor Vladimirescu Ave., 300173 Timisoara, Romania; 5 Faculty of Food Engineering, Banat’s University of Agricultural Sciences and Veterinary Medicine, King Michael I of Romania” From Timișoara, Calea Aradului No. 119, 300645 Timișoara, Romania

**Keywords:** Medical research, Dentistry, Diagnosis, Medical imaging, Therapeutics, Oral diseases

## Abstract

A major factor that contributes to dental malocclusions is represented by the positioning of the mandible. Considering the existing interconnections between the craniocervical and craniomandibular systems it is interesting to assess how changes in one system can influence the other, thus establishing a pattern in terms of certain cephalometric landmarks that orthodontists could consider when diagnosing and evaluating an orthodontic case. Therefore, the aim of this study was to investigate the connections between cervical posture, head position, hyoid bone position in orthodontic patients with different skeletal patterns. 45 lateral cephalometric radiographs were analyzed. Skeletal class and vertical growth were the main elements that were considered when classifying patients. Craniofacial and Cervical landmarks were determined on the cephalograms, from which lines and angles resulted which were considered relevant in our study. Correlations between cephalometric variables of the patients were determined. there were some statistically significant changes identified concerning craniocervical posture and hyoid bone position between the patients in the following parameters: H-Rgn, OPT/HOR, CVT/HOR, OPT/SN, CVT/SN, H-SN. The results obtained allowed us to conclude that there were some differences at the skeletal level of the sample of patients studied. The findings are indicating that there is a close relationship between, mandible position, cervical- and head posture and the hyoid bone. The information obtained in this study could help to better understand the development of malocclusions, and to improve the orthodontic diagnostic and treatment plan.

## Introduction

The craniofacial complex is an extremely elaborated structure at which the forces applied during the orthodontic treatment take place. Taking into consideration that there are correlations between the craniocervical and craniomandibular systems, it is important to determine whether anatomical variations in one of these systems can cause changes in the other and vice versa, as well as to establish if these elements can be useful during orthodontic diagnosis and treatment planning^1^. Previous studies show the close relationship between the craniomandibular and craniocervical systems, both having the potential to influence each other^2–6^.

An anatomical element that is related both to the craniocervical system and the craniomandibular system, whose role during orthodontic treatment has not yet been fully elucidated, is the hyoid bone. The hyoid bone is a key component in the musculoskeletal system of the craniofacial complex^7^. It plays an important role in the physiological development of functions in the head and neck region such as food intake, breathing and speech, its position adapting accordingly to changes in head posture^8^. The hyoid bone is an anatomical element with an important role in the positioning of the head and neck. Previous research has shown that the hyoid bone can adapt to changes in the anteroposterior direction of the head posture and that changes of the mandible position are related with changes of the hyoid bone^9,10^. It is known that there are differences in the physiological development of functions in individuals with variations regarding their craniofacial anatomical relationships^11^. Normal functions such as mastication and breathing influence the neuromuscular system, which in turn influences the cranial posture. Thus, it was observed that the flexion and extension movements of the head that modify the Natural head position (NHP) are associated with morphological patterns^12^. Previous studies demonstrated that changes in cephalic posture related to some functional alterations such as oral respiration influences the growing patient, consequently leading to the development of typical malocclusive patterns such as mandibular retrognathia, increased gonial and craniocervical angles and causing modifications in the lingual-mandibular-hyoid complex. Therefore, in oral breathing patients, cranial extensions are frequently observed. These postural changes could be a compensation for nasal airway inadequacy^13^.

As age advances, the hyoid bone may descend to lie at the level of the fourth cervical vertebra. Studies show that there is a relationship between cervical posture and dental occlusion. Patients with a class II Angle malocclusion tend to have an exaggerated kyphosis of the cervical vertebras compared to patients with normal dental occlusion^14^. This extended head posture is accompanied by a change in the resting position of the mandible and subsequently in an increase in the occlusal freeway space^15^.

A paraclinical investigation that can highlight the relationships between cervical posture and dental malocclusion is represented by the lateral cephalogram, which is a specialized radiographic technique concerned with imaging the craniofacial region in a standardized and reproductible manner^16^.

Many cephalometric analyses are available these days, but only few take into consideration the relationship established between craniocervical posture and it’s connection to dental malocclusion.

Thus, aim of this study was to investigate the characteristics of craniocervical morphology in 3 groups of patients divided according to the ANB angle and according to their vertical growth pattern before starting orthodontic treatment.

## Materials and methods

### Study design

This was an observational and retrospective study. The sample consisted of 45 lateral cephalograms from patients aged between 25 and 30, thus considering that skeletal growth has reached maturity. Patients who have undergone any orthodontic treatment in the past were excluded from the study. Also, patients who reported systemic illnesses, musculoskeletal disorders, traumatic injuries, symptoms of TMD, complex dental restorations or symptoms of attrition were excluded. To be included in the study patients had to have lateral and anterior photographs in a natural head position (NHP) and the teleradiography had to be of good quality (also in natural head position) and to highlight at least 4 cervical vertebrae.

Ethical approval was guaranteed by the Ethics Committee at Victor Babes University of Medicine and Pharmacy Timisoara:” Aviz CECS Nr. 13/26.03.2021”. All methods were performed in accordance with the relevant guidelines and regulations.

The method for obtaining the lateral cephalogram in NHP was performed as described by Meiyappan et al.^17^ The lateral photographs which were also performed in an NHP, were used for superimposition, and allowed for the confirmation of a natural head position. The cephalometric software used for teleradiograph analysis with lateral photographs superimposition in NHP were performed with AudaxCeph® software (AudaxCeph Ultimate Ver 6.3.11.4346, Ljublijana, Slovenia)®.

The patients were divided into three groups, according to the ANB angle which measures the position of the maxilla in relation to the mandible in sagittal plane indicating the skeletal pattern of the patients: class I (0°–4°), class II (> 4°), class III (< 0°). In each sagittal group there were the following samples: 18 for class I; 16 for class II; 11 for class III. Also, vertical growth pattern was considered when assessing hyoid bone position. In this sense, patients were divided into 3 other categories: normodivergent (22°–28°), hyper-divergent (> 28°) and hypodivergent (< 22°), depending on the value of the FMA angle. The sample sizes for the classification regarding the vertical growth pattern were the following: 21 samples normodivergent, 14 samples hyper-divergent and 10 samples hypodivergent.

In order to evaluate the reliability of the cephalometric measurements, these were performed twice by Dr. Vlad-Tiberiu Alexa (Orthodontics and Dentofacial Orthopedics Specialist) at a one-week interval. The averages of these values found in the two measurements were used for assessment. The measurement errors were determined with respect to these averages. Fourteen days after the measurements, twelve lateral radiographs were randomly chosen for examining the error made between the two periods of tracings. The correlation coefficients between the measurement errors where greater than 0.95, demonstrating an excellent reliability.

**Figure 1:**
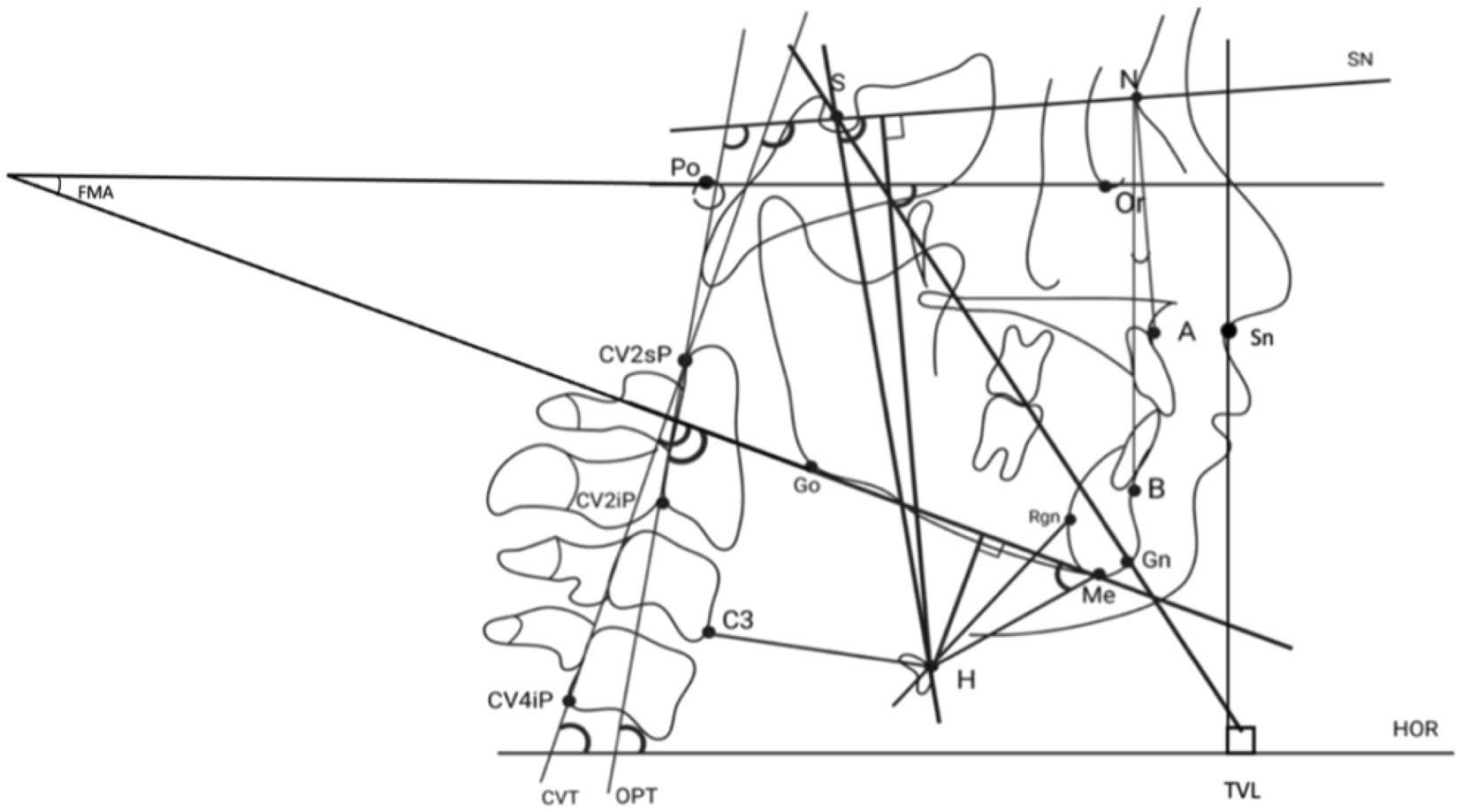
Anatomical landmarks.

Figure 1 represents the anatomical landmarks from which lines and angles resulted. The cephalometric landmarks and their references are represented in the Table 1.

**Table 1:** Cephalometric landmarks and their references.

**Cephalometric points**	
Point A	The most posterior point on the curvature of the anterior nasal spine
Point B	The most posterior point on the profile of the mandibular alveolar process
Nasion (N)	The most anterior point on the fronto-nasal suture in the mid-sagittal plane
Sella (S)	The geometric center of the contour of the Sella turcica
Porion (Po)	Most superior point of outline of external auditory meatus
Orbitale (Or)	Most inferior point on margin of orbit
C3	The most anterior and inferior point of the third cervical vertebra
Hyoid (H)	The most anterior and superior point on the body of the hyoid bone
Gonion (Go)	The most posterior, lateral and inferior point on the outer face of the mandibular angle
Menton (Me)	The lowest point on the chin symphysis
Gnathion (Gn)	The most anterior and inferior point of the chin
Retrognation (Rgn)	The most posterior point of the chin symphysis
Cv2sp	The most posterior and superior point of the body of the second cervical vertebra
Cv2ip	The most infero-posterior point of 2nd vertebra
Cv4ip	The most posterior and inferior point of the body of the fourth cervical vertebra
Subnasale (Sn)	The point where the nasal septum and the upper lip meet in the midsagittal plane
**Cephalometric lines and planes used**	
Go-Me (ML)	The mandibular plane
S–N (SN)	The anterior cranial base plane
Po-Or (FK)	Frankfort horizontal plane
Y-axis	A line connecting S to Gn
OPT	Odontoid process line. A line through cv2ip and cv2sp
CVT	Cervical vertebrae tangent. A line through cv2sp and cv4ip
TVL	True vertical line. Is a perpendicular line passing through the subnasale point (Sn)
HOR	True horizontal line. A horizontal line drawn through TVL
**Cephalometric linear measurements**	
H-ML	Perpendicular from H to the mandibular plane
H-C3	Linear distance from H to C3
H-RGn	Linear distance between H and RGn
H-SN	Linear distance along a perpendicular from H to the S–N plane
**The cephalometric angular measurements**	
SNA	The angle from sella to nasion to point A
SNB	The angle from sella to nasion to point B
ANB	The angle joining point A to nasion (N) to point B, SNA-SNB difference
FMA	The angle between Frankfurt plane and mandibular plane. This angle determines the skeletal pattern of the patients in vertical plane
H/SN	The angle from Nasion to Sella to Hyoidale
H/ML	The angle from Gonion to Menton to Hyoidale
Y-axis to Frankfurt horizontal plane	An estimate of mandibular growth direction
OPT/HOR	The angle between OPT and the horizontal line. The posterior tangent to the odontoid process through CV2sp to the true horizontal
CVT/HOR	The angle between CVT and HOR. The posterior tangent to the odontoid process through CV4ip to the true horizontal
OPT/SN	The angle between anterior cranial base and the odontoid process tangent (OPT). Head position in relation to the 2nd cervical vertebrae
CVT/SN	The angle between NSL and the cervical vertebrae tangent. Head position in relation to the 2nd and 4th cervical vertebrae
OPT/ML	Mandibular base inclination upon cervical column. Downward angle between Go-Me and OPT line
CVT/ML	Mandibular base inclination upon cervical column. Downward angle between Go-Me and CVT line

### Statistical analysis

The mean values and standard deviations of all replicates were calculated using GraphPad Prism (v.5.0 software, Manufacture, San Diego, CA, USA). Differences between means were analyzed with a one-way ANOVA, followed by multiple comparison analysis using the Duncan test (two-sample assuming equal variances). Differences were considered significant when p-values < 0.05. Correlations between variable was performed using Microsoft Excel 2010. Duncan tests, linear correlations between variable, principal components analysis and cluster analysis were performed using Statistica (v.12, TIBCO Software, Palo Alto, California, USA).

## Results

### Cephalometric differences between the groups

**Figure 2:**
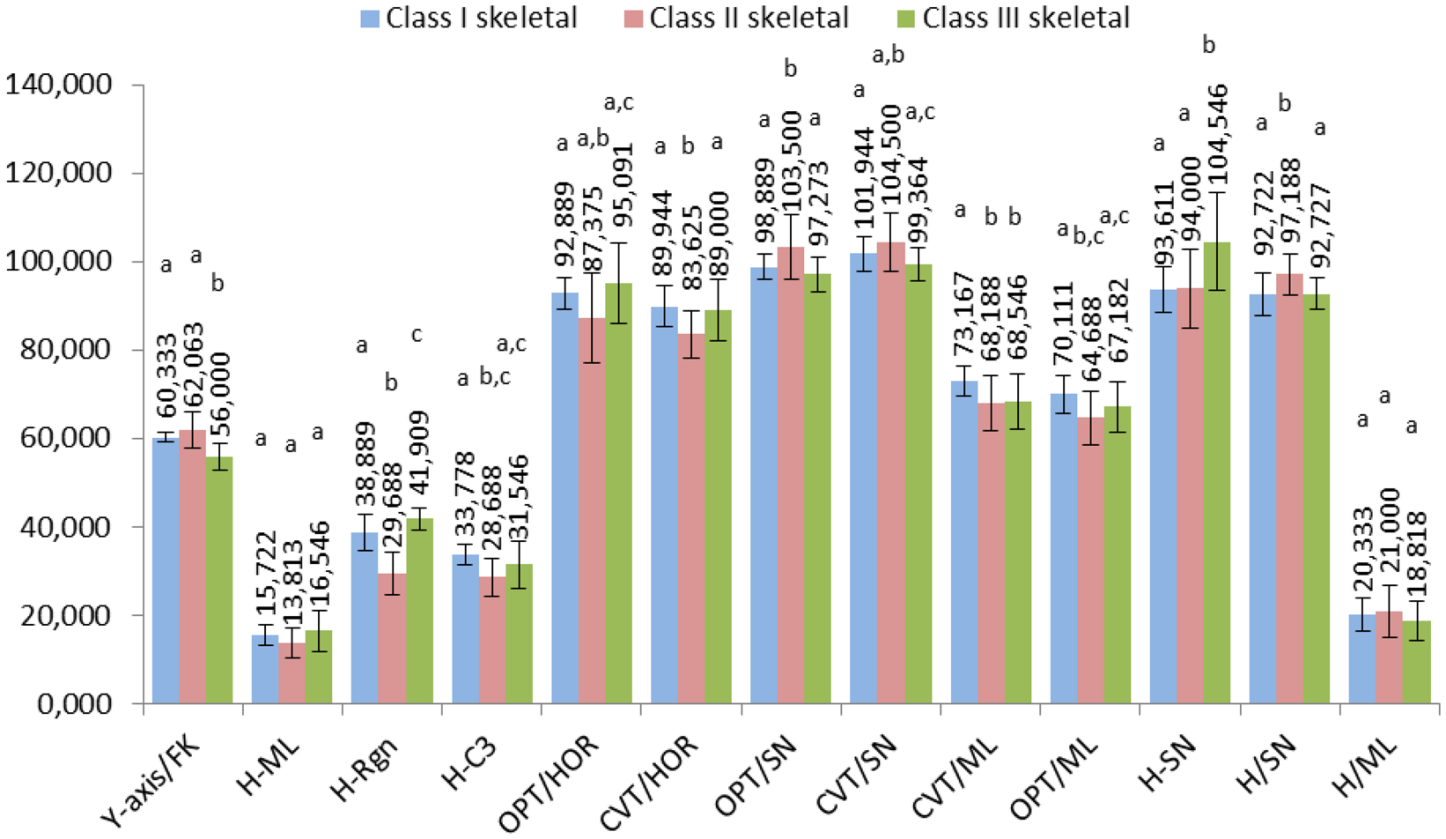
The average values of the determined parameters for different skeletal classes. Different letter in the row indicates significant differences (p < 0.05) between values according to the Duncan test.

**Figure 3:**
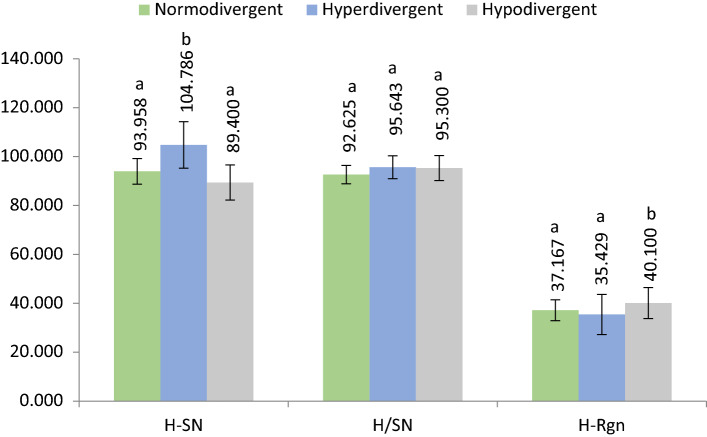
The average values of the determined parameters assessing the position of the hyoid bone between patients with different vertical growth patterns. Different letter in the row indicates significant differences (p < 0.05) between values according to the Duncan test.

Hyoid bone, head position and craniocervical position in relation to certain landmarks were analyzed and the values were statistically interpreted using the Duncan test (Fig. 2). Figure 3 represents average values of the determined parameters assessing the position of the hyoid bone between patients with different vertical growth patterns.

### Correlations between variables

The analysis of correlation (Table 2) highlights a strong (r > 0.7) positive correlation between pairs I_CVT/HOR/ I_Y-axis/FK, (r = 0.737), a moderate (r > 0.5) positive correlation between the pairs I_H/ML and I_ANB (r = 0.526), I_OPT/HOR and I_H-C3 (r = 0.682). We determined mutual correlations between individual variables, which can prove dependence between parameters. The highest correlation coefficient values were determined for the I_CVT/HOR/ I_Y-axis/FK, where we can presume their mutual connection.

**Table 2:** Matrix of Pearson linear correlation (class I).

Variable	Pearson correlations (class I)													
	I_ANB	I_Y-axis/FK	I_H-ML	I_H-Rgn	I_H-C3	I_OPT/HOR	I_CVT/HOR	I_OPT/SN	I_CVT/SN	I_CVT/ML	I_H-SN	I_OPT/ML	I_H/SN	I_H/ML
I_ANB	1.000	**0.498**	0.335	0.049	− 0.395	**0.469**	0.255	− 0.167	0.080	− 0.061	− 0.440	0.115	0.008	**0.526**
I_Y-axis/FK	**0.498**	1.000	0.195	− 0.131	− 0.138	0.031	**0.737**	− 0.199	− 0.159	0.083	− 0.429	0.395	0.228	0.327
I_H-ML	0.335	0.195	1.000	0.141	0.436	− 0.357	0.311	− 0.073	− 0.122	0.054	− 0.189	0.314	0.158	0.250
I_H-Rgn	0.049	− 0.131	0.141	1.000	0.073	− 0.104	0.057	0.147	− 0.058	− 0.048	− 0.272	− 0.161	− 0.187	− 0.177
I_H-C3	− 0.395	− 0.138	0.436	0.073	1.000	**− 0.682**	0.219	0.118	0.195	0.169	0.306	0.125	− 0.069	− 0.094
I_OPT/HOR	**0.469**	0.031	− 0.357	− 0.104	− **0.682**	1.000	− 0.030	− 0.112	− 0.037	− 0.142	− 0.348	− 0.057	− 0.303	0.140
I_CVT/HOR	0.255	**0.737**	0.311	0.057	0.219	− 0.030	1.000	− 0.046	− 0.186	− 0.082	− 0.382	0.403	0.178	0.352
I_OPT/SN	− 0.167	− 0.199	− 0.073	0.147	0.118	− 0.112	− 0.046	1.000	**0.527**	− 0.181	0.076	− 0.105	0.290	− 0.167
I_CVT/SN	0.080	− 0.159	− 0.122	− 0.058	0.195	− 0.037	− 0.186	**0.527**	1.000	0.073	0.133	− 0.400	0.079	0.060
I_CVT/Mp	− 0.061	0.083	0.054	− 0.048	0.169	− 0.142	− 0.082	− 0.181	0.073	1.000	0.447	− 0.057	− 0.092	− 0.103
I_OPT/ML	− 0.440	− 0.429	− 0.189	− 0.272	0.306	− 0.348	− 0.382	0.076	0.133	0.447	1.000	− 0.380	0.097	− 0.120
I_H-SN	0.115	0.395	0.314	− 0.161	0.125	− 0.057	0.403	− 0.105	− 0.400	− 0.057	− 0.380	1.000	− 0.048	0.292
I_H/SN	0.008	0.228	0.158	− 0.187	− 0.069	− 0.303	0.178	0.290	0.079	− 0.092	0.097	− 0.048	1.000	0.254
I_H/ML	**0.526**	0.327	0.250	− 0.177	− 0.094	0.140	0.352	− 0.167	0.060	− 0.103	− 0.120	0.292	0.254	1.000

Significant values are in bold.

**Figure 4:**
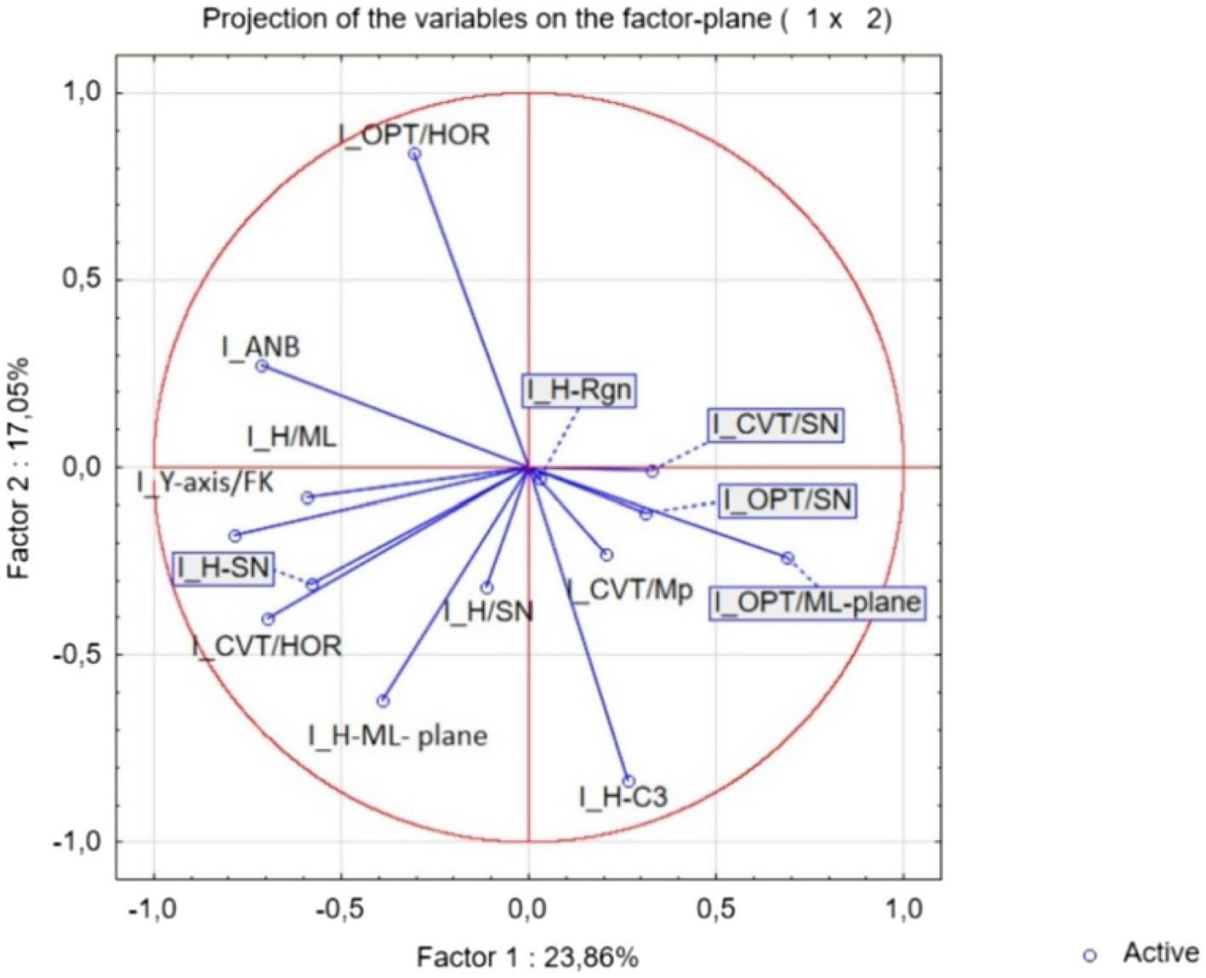
Projection of the parameters (cephalometric measurements) from the class I patients on the plane spanned by the first and second principal components.

**Figure 5:**
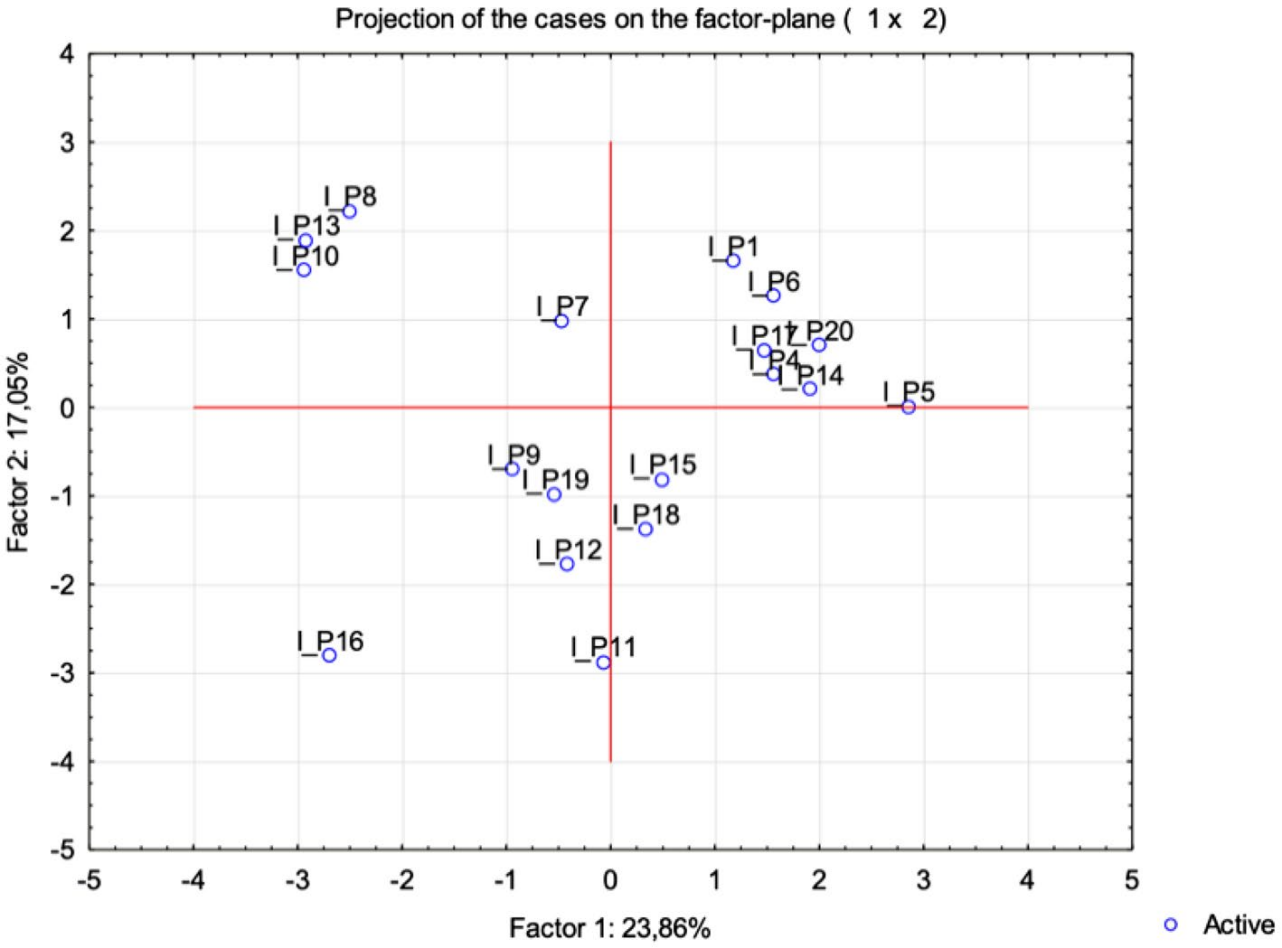
Projection of the cases for class I on the plane spanned by the first and second principal components.

**Figure 6:**
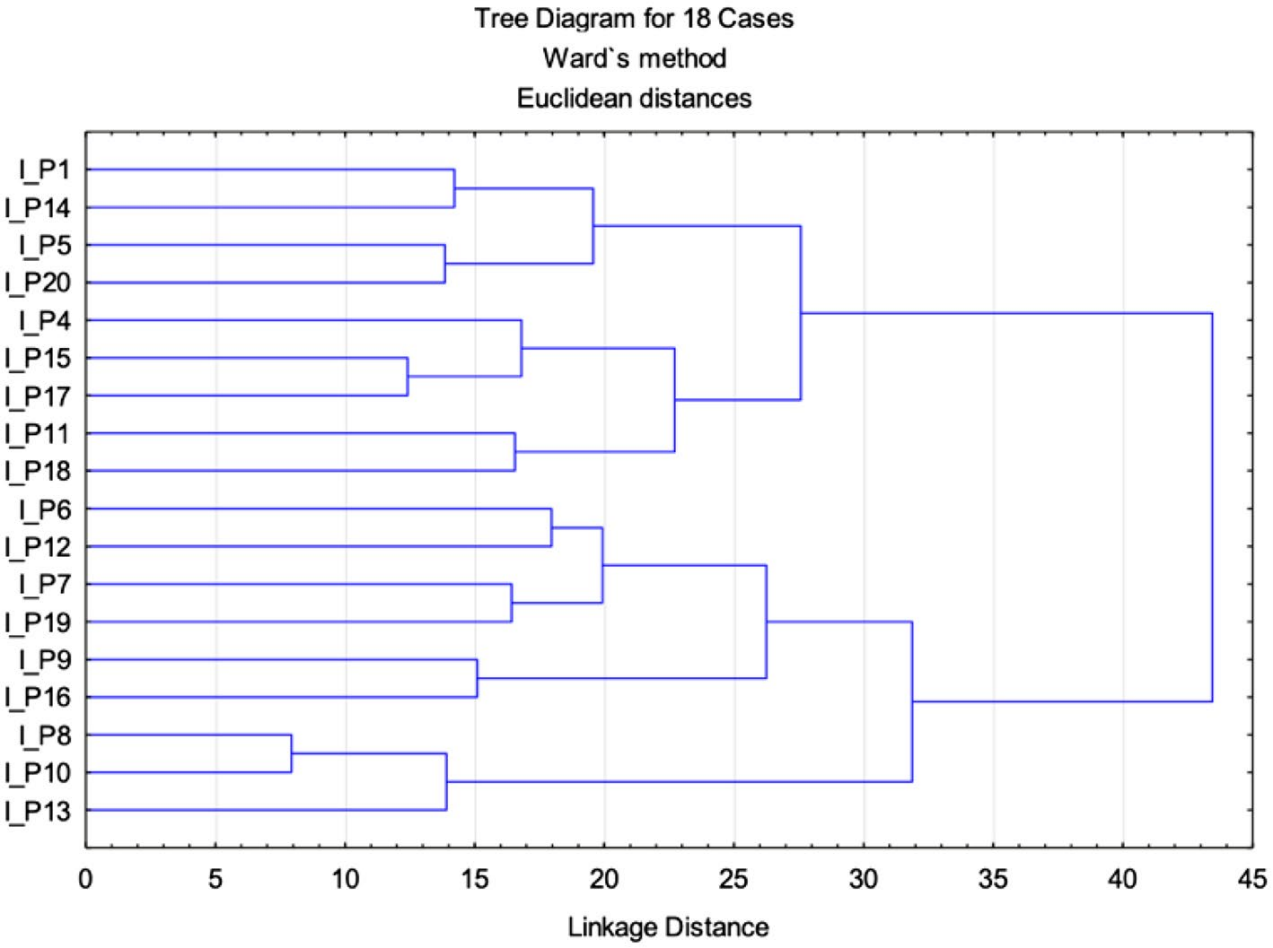
Cluster dendrogram of class I.

In addition, the multi-parametric statistical evaluation method using PCA (Figs. 4 and 5), and CA (Fig. 6) were used for further data analysis. Based on a linear correlation matrix, PCA was applied to the mean values of the measured traits to study which parameters contributed the most to total data variation.

The PCA produced 14 components. The first two principal components accounted for 23.86% and 17.05% (a total of 40.90%) of the variance, respectively. The most important variables integrated in the first component were: I_OPT/ML which was positively correlated with this component; on the other hand, I_ANB, I_Y-axis/FK, I_CVT/HOR, I_H-SN, I_H/ML were negatively correlated with this first component. The second component was positively correlated with I_OPT/HOR and negatively correlated with I_H-ML and I_H-C3 (Fig. 4).

The similarity among class I patients was examined when each sample was plotted using the first and second principal components, which retained 40.90% of the total variance (Fig. 4) and showed a clustering tendency, leading us to carry out correspondence analysis and depict the case through a simplified dendrogram plot. We can clearly identify class I patients with distant values by this data presentation. We were then able to put forward a dendrogram using CA, which divides class I patients into five representative clusters based on the correlation of their determined parameters.

Clusters classify individual patients into five groups based on similar characteristics: Group 1: I_P1, I_P14, I_P15, I_P20; Group 2: I_P4, I_P15, I_P17; Group 3: I_P6, I_12, I_P7, I_P19; Group 4: I_P9 and I_P16; and Group 5: I_P8, I_P10 and I_P13.

The analysis of correlation between parameters recorded in the II class (Table 3) highlights a strong (r > 0.7) positive correlation between pairs II_CVT/ML/ II_OPT/ML, (r = 0.701), a moderate (r > 0.5) positive correlation between the pairs II_H/ML and II_H-SN (r = 0.584), II_H/SN and II_OPT/SN (r = 0.583), II_H/ML and II_H-ML (r = 0.606), II_OPT/ML and II_H-Rgn (r = 0.545), II_OPT/ML and II_CVT/SN (r = 0.609), II_CVT/SN and II_H-C3 (r = 0.509), II_H-Rgn and II_Y-axis/FK (r = 0.520). We determined mutual correlations between individual variables, which can prove dependence between parameters. The highest correlation coefficient values were determined for the I_CVT/HOR/ I_Y-axis/FK, where we can presume their mutual connection.

**Table 3:** Matrix of Pearson linear correlation (class II).

Variable	Pearson correlations (class II)													
	II_ANB	II_Y-axis/FK	II_H-ML	II_H-Rn	II_H-C3	II_OPT/HOR	II_CVT/HOR	II_OPT/SN	II_CVT/SN	II_CVT/ML	II_OPT/ML	II H-SNI_H-SN	II_H/SN	II_H/ML
II_ANB	1.000	0.301	− 0.171	− 0.079	0.148	− 0.102	− 0.255	− 0.170	− 0.217	0.106	− 0.002	− 0.301	− 0.195	− 0.093
II_Y-axis/ K	0.301	1.000	0.112	− **0.520**	0.429	0.097	− 0.169	− 0.054	0.088	− 0.177	− 0.074	0.299	− 0.179	0.277
II_H-ML	− 0.171	0.112	1.000	0.274	− 0.212	− 0.192	− 0.415	0.265	0.179	0.074	− 0.012	0.327	0.128	**0.606**
II_H-Rgn	− 0.079	− **0.520**	0.274	1.000	0.024	− 0.181	− 0.102	0.071	0.287	0.424	**0.545**	− 0.062	− 0.009	− 0.075
II_H-C3	0.148	0.429	− 0.212	0.024	1.000	− 0.070	− 0.163	0.239	**0.509**	0.373	0.442	0.165	0.227	0.067
II_OPT/HOR	− 0.102	0.097	− 0.192	− 0.181	− 0.070	1.000	0.243	− 0.151	0.287	− 0.107	0.080	0.131	− 0.348	0.228
II_CVT/HOR	− 0.255	− 0.169	− 0.415	− 0.102	− 0.163	0.243	1.000	− 0.101	− 0.073	− 0.288	0.000	− 0.135	− 0.150	− 0.444
II_OPT/SN	− 0.170	− 0.054	0.265	0.071	0.239	− 0.151	− 0.101	1.000	0.496	0.112	0.212	0.057	**0.583**	0.101
II_CVT/SN	− 0.217	0.088	0.179	0.287	**0.509**	0.287	− 0.073	0.496	1.000	0.438	**0.609**	0.405	0.263	0.260
II_CVT/ML	0.106	− 0.177	0.074	0.424	0.373	− 0.107	− 0.288	0.112	0.438	1.000	**0.701**	− 0.129	0.375	− 0.165
II_OPT/ML	− 0.002	− 0.074	− 0.012	**0.545**	0.442	0.080	0.000	0.212	**0.609**	**0.701**	1.000	− 0.061	0.175	− 0.134
II_H-SN	− 0.301	0.299	0.327	− 0.062	0.165	0.131	− 0.135	0.057	0.405	− 0.129	− 0.061	1.000	0.081	**0.584**
II_H/SN	− 0.195	− 0.179	0.128	− 0.009	0.227	− 0.348	−0.150	**0.583**	0.263	0.375	0.175	0.081	1.000	0.182
II_H/ML	− 0.093	0.277	**0.606**	− 0.075	0.067	0.228	− 0.444	0.101	0.260	− 0.165	− 0.134	**0.584**	0.182	1.000

Significant values are in bold.

In addition, the multi-parametric statistical evaluation method using PCA (Figs. 8 and 9), and CA (Fig. 10) were used for further data analysis.

**Figure 7:**
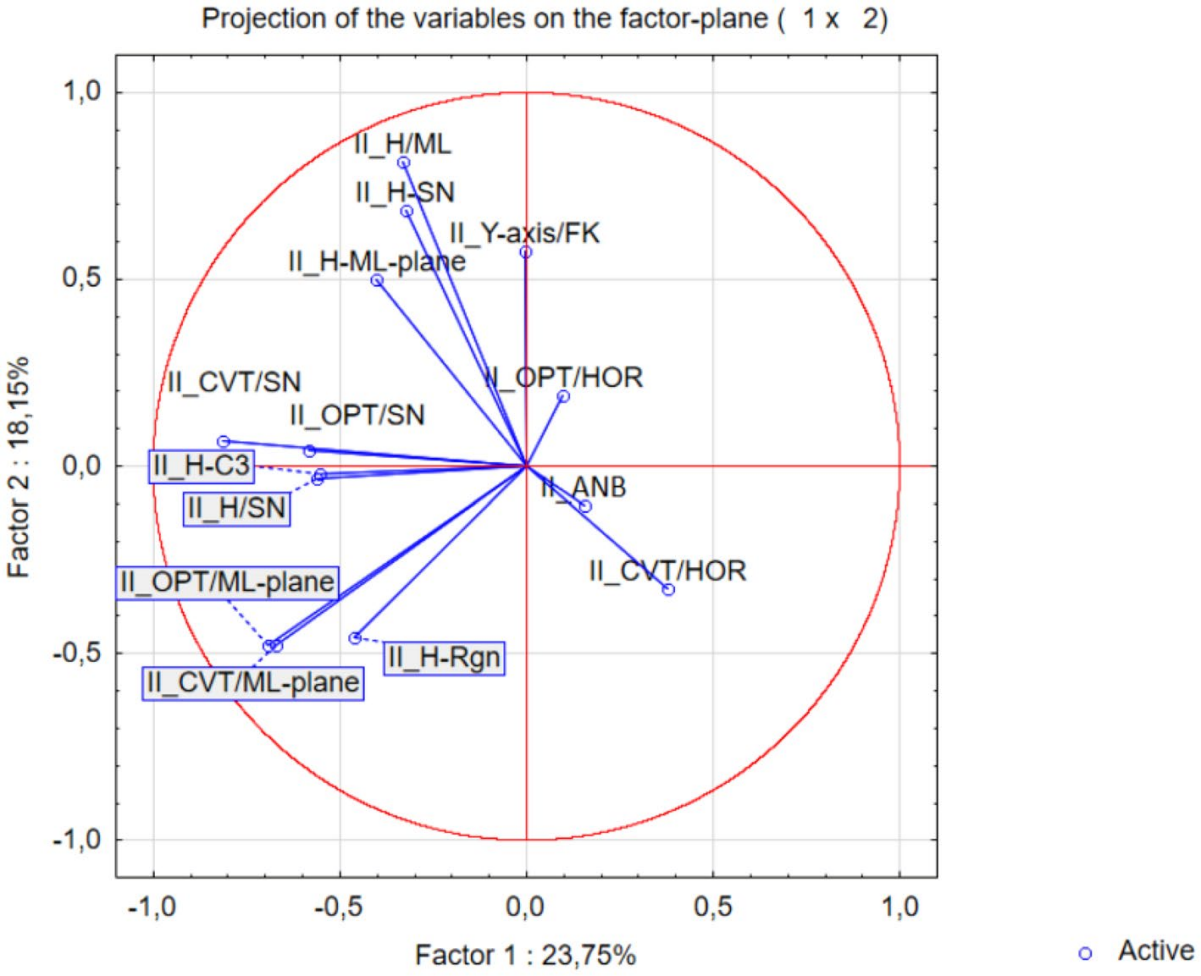
Projection of the parameters (cephalometric measurements) from the class II patients on the plane spanned by the first and second principal components.

With the aim of studying which parameters assisted the most the total data variation, PCA was applied for the mean values of the determined traits, based on a linear correlation matrix. The PCA produced 14 components. The first two principal components accounted for 23.75% and 18.15% (a total of 41.90%) of the variance, respectively. The most important variables integrated in the first component were II_H-Rgn, II_H-C3, II_OPT/SN, II_CVT/SN, II_CVT/ML, II_OPT/ML and II_H/SN which were negatively correlated with this first component. The second component was positively correlated with II_Y-axis/FK, II_H-ML, II_H-SN and II_H/ML (Fig. 7).

**Figure 8:**
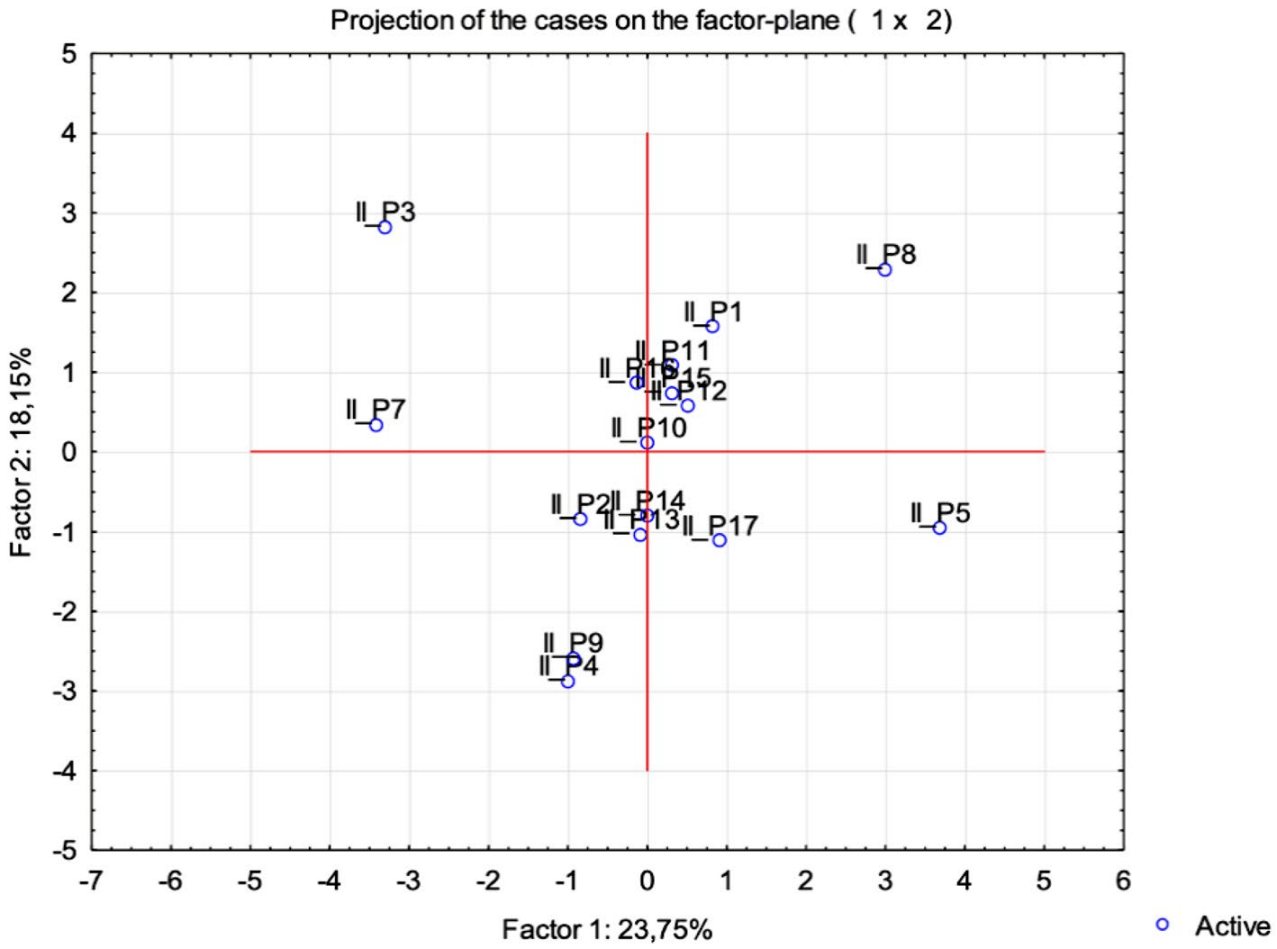
Projection of the cases for class II on the plane spanned by the first and second principal components.

The similarity among class II patients was examined when each sample was plotted using the first and second principal components, which retained 41.90% of the total variance (Fig. 8) and showed a clustering tendency, leading us to carry out correspondence analysis and depict the case through a simplified dendrogram plot.. We can clearly identify class II patients with distant values by this data presentation. We were then able to put forward a dendrogram using CA, which divides class I patients into five representative clusters based on the correlation of their determined parameters.

**Figure 9:**
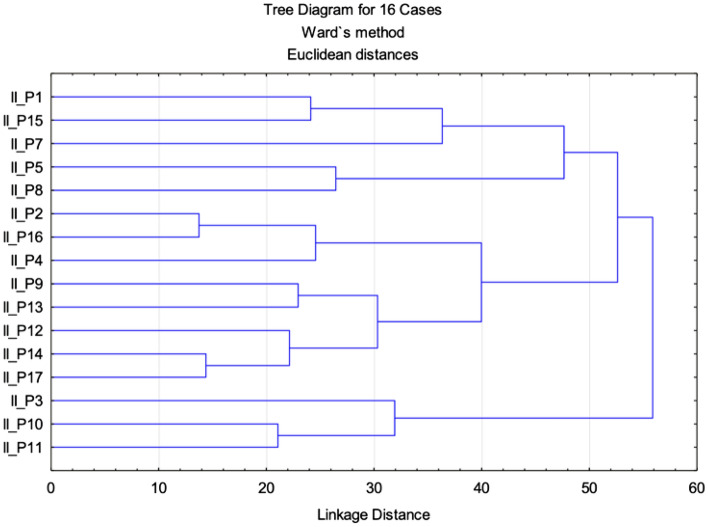
Cluster dendrogram of class II.

Clusters classify individual patients into five groups based on similar characteristics: Group 1: II_P1, II_P15, II_P7; Group 2: II_P5, II_P8; Group 3: II_P2, II_16, II_P4; Group 4:II_P9, II_P9, II_P12, II_P14 and II_P17; and Group 5: II_P3, II_P10 and II_P11 (Fig. 9).

Regarding the parameters of III class, the analysis of correlation (Table 4) highlights a strong (r > 0.7) positive correlation between pairs: III_CVT/ML and III_OPT/HOR (r = 0.633), III_OPT/SN (r = 0.744), III_H-SN/III_H-Rgn, (r = 0.705), III_H-SN and III_H-C3 (r = 0.728), III_OPT/SN and III_CVT/ML (r = 0.744), a moderate (r > 0.5) positive correlation between the pairs: III_CVT/SN and III_ANB (r = 0.623), III_H-SN and III_OPT/HOR (r = 0.633); III_H-C3 and III_CVT/SN (r = 0.633); III_H/ML and III_H-ML (r = 0.652), III_CVT/HOR and III_opt/HOR (r = 0.642).

**Table 4:** Matrix of Pearson linear correlation (class III).

Variable	Correlations (statistics descriptive class III)													
	III_ANB	III_Y-axis/FK	III_H-ML	III_H-Rgn	III_H-C3	III_OPT/HOR	III_CVT/HOR	III_OPT/SN	III_CVT/SN	III_CVT/ML	III_OPT/ML	III_H-SN	III_H/SN	III_H/ML
III_ANB	1.000	0.116	0.162	− 0.361	0.397	0.182	− 0.086	0.551	**0.623**	0.433	0.023	0.090	0.150	*0.212*
III_Y-axis/FK	0.116	1.000	− 0.231	− 0.112	0.018	0.244	0.291	− 0.155	− 0.178	− 0.499	0.269	− 0.109	− 0.371	− *0.190*
III_H-ML	0.162	− 0.231	1.0.00	0.455	0.328	− 0.116	− 0.157	0.198	0.084	0.277	− 0.120	0.588	0.311	***0.652***
III_H-Rgn	− 0.361	− 0.112	0.455	1.000	0.337	− 0.395	− 0.162	0.254	− 0.090	0.047	0.263	**0.705**	− 0.147	*0.483*
III_H-C3	0.397	0.018	0.328	0.337	1.000	− 0.252	0.010	0.233	**0.633**	0.203	0.182	**0.728**	− 0.216	*0.408*
III_OPT/HOR	0.182	0.244	− 0.116	− 0.395	− 0.252	1.000	**0.642**	0.109	− 0.491	0.188	0.490	**− 0.633**	0.145	*0.31.3*
III_CVT/HOR	− 0.086	0.291	− 0.157	− 0.162	0.010	**0.642**	1.000	− 0.014	− 0.308	0.141	0.531	− 0.193	0.012	*0.336*
III_OPT/SN	0.551	− 0.155	0.198	0.254	0.233	0.109	− 0.014	1.000	0.231	**0.774**	0.416	0.240	− 0.103	*0.568*
III_CVT/SN	**0.623**	− 0.178	0.084	− 0.090	**0.633**	− 0.491	− 0.308	0.231	1.000	0.314	− 0.139	0.477	0.174	− *0.036*
III_CVT/ML	0.433	− 0.499	0.277	0.047	0.203	0.188	0.141	**0.774**	0.314	1.000	0.428	0.103	0.224	*0.598*
III_OPT/ML	0.023	0.269	− 0.120	0.26.3	0.182	0.490	0.531	0.416	− 0.139	0.428	1.000	− 0.079	− 0.092	0.489
III_H-SN	0.090	− 0.109	0.588	**0.705**	**0.728**	− **0.633**	− 0.193	0.240	0.477	0.103	− 0.079	1.000	− 0.090	0.435
III_H/SN	0.150	− 0.371	0.311	− 0.147	− 0.216	0.145	0.012	− 0.103	0.174	0.224	− 0.092	− 0.090	1.000	0.221
III_H/ML	0.212	− 0.190	**0.652**	0.483	0.408	0.313	0.336	0.568	− 0.036	0.598	0.489	0.435	0.221	1.000

Significant values are in bold.

The highest correlation coefficient values were determined for the III_CVT/HOR/ III_Y-axis/FK, where we can presume their mutual connection.

**Figure 10:**
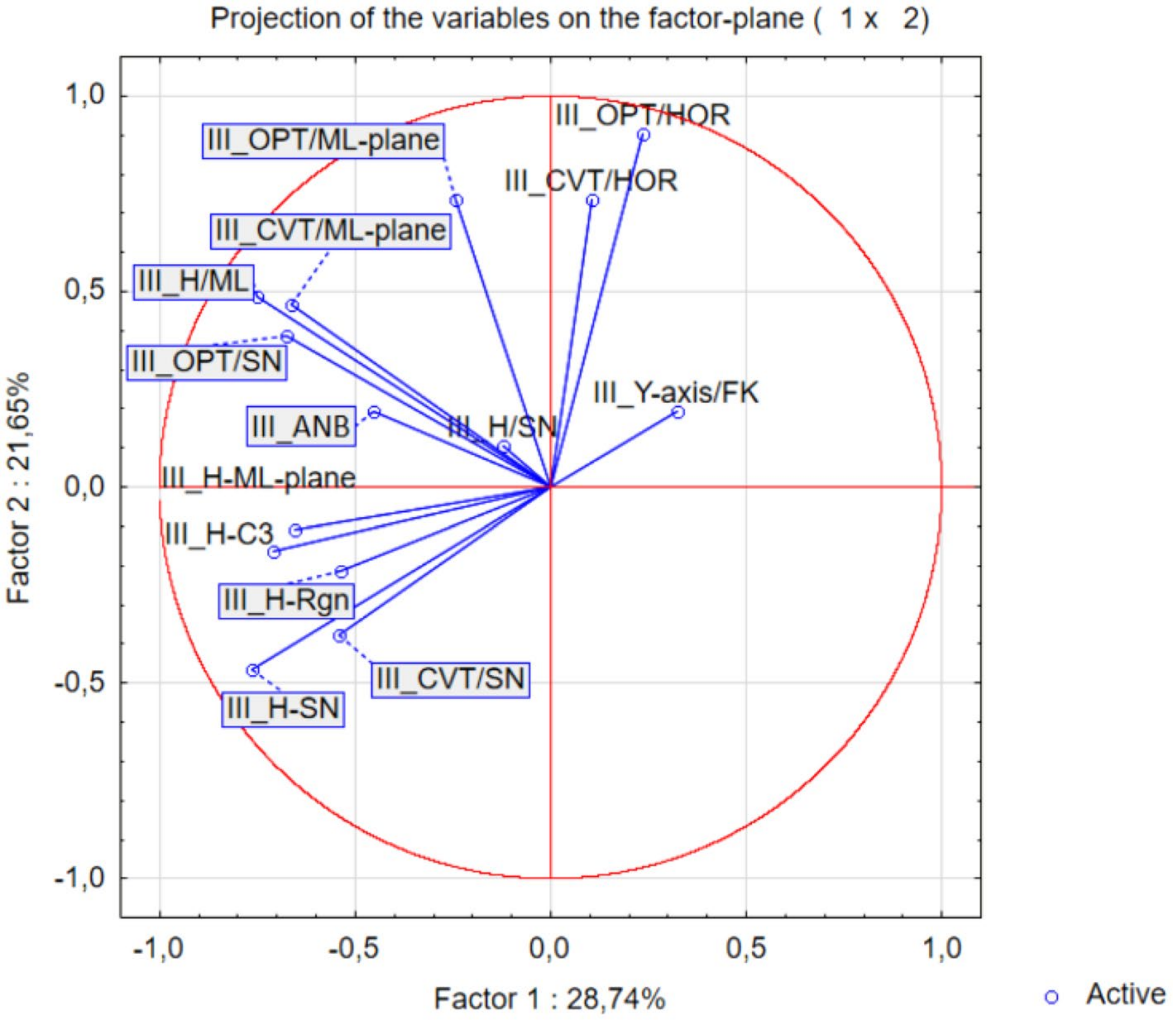
Projection of the parameters (cephalometric measurements) from the class III patients on the plane spanned by the first and second principal components.

**Figure 11:**
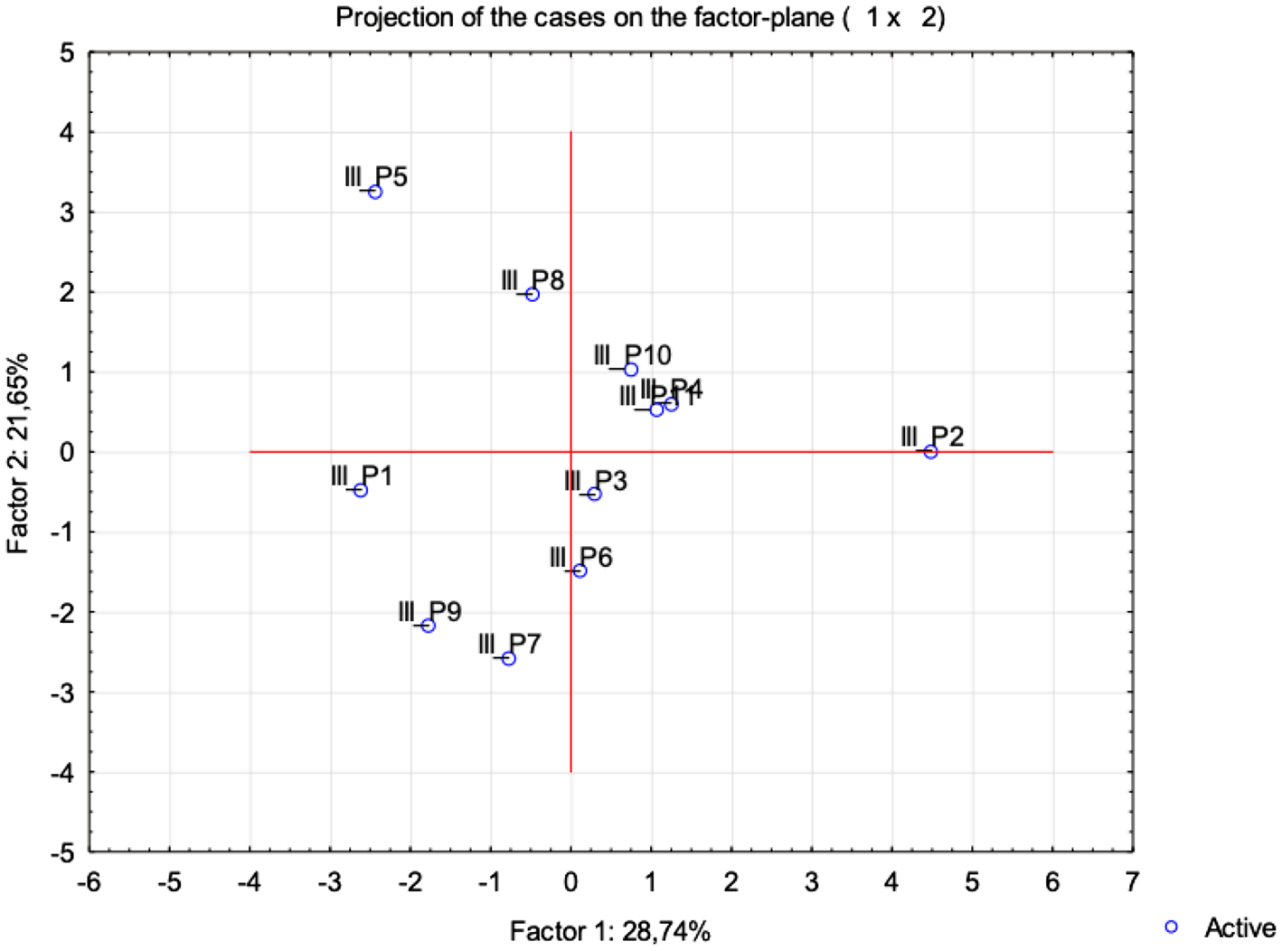
Projection of the cases for class III on the plane spanned by the first and second principal components.

**Figure 12:**
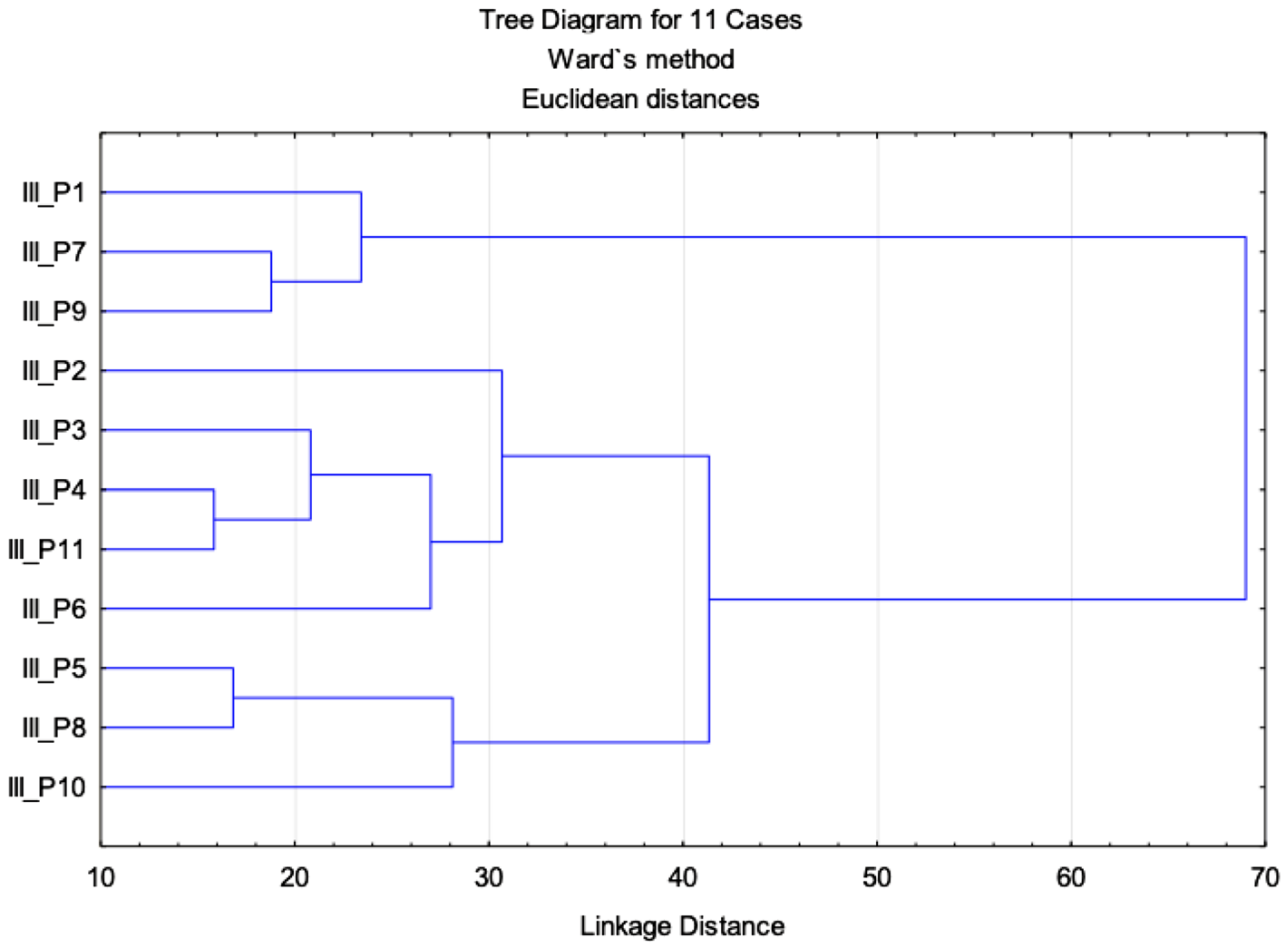
Cluster dendrogram of class III.

In addition, the multi-parametric statistical evaluation method using PCA (Figs. 10 and 11) and CA (Fig. 12) were used for further data analysis.

Based on a linear correlation matrix, PCA was applied to the mean values of the measured traits to study which parameters contributed the most to total data variation. The PCA produced 10 components. The first two principal components accounted for 28.74% and 21.65% (a total of 50.39%) of the variance, respectively. The most important variables integrated in the first component were III_H-ML, III_H-Rgn, III_H-C3, III_OPT/SN, III_CVT/SN, III_CVT/ML, III_H-SN and III_H/ML which were negatively correlated with this first component. The second component was positively correlated with III_OPT/HOR, III_CVT/HOR and III_OPT/ML (Fig. 10).

The similarity among class III patients was examined when each sample was plotted using the first and second principal components, which retained 50.39% of the total variance (Fig. 11). This led us to perform CA and represent the case by a simplified dendrogram plot. Using CA, we were able to suggest a dendrogram that divides class III patients into three representative clusters based on the mutual similarity of their measured parameters (Fig. 11).

Clusters classify individual patients into three groups based on similar characteristics: Group 1: III_P1, III_P9, III_P7; Group 2: III_P2, III_P3, III_P4, III_11, III_6 (Fig. 12).

Regarding the parameters of III class, the analysis of correlation (Table 4) highlights a strong (r > 0.7) positive correlation between pairs: III_CVT/ML and III_OPT/SN (r = 0.774), III_H-SN / III_H-Rgn, (r = 0.705), III_H-SN and III_H-C3 (r = 0.728), a moderate (r > 0.5) positive correlation between the pairs: III_OPT/HOR and III_CVT/HOR (r = 0.642), III_CVT/SN and III_ANB (r = 0.623), III_H-C3 and III_CVT/SN (r = 0.633); III_H/ML and III_H-ML (r = 0.652), III_CVT/HOR and III_OPT/HOR (r = 0.642).

The highest correlation coefficient values were determined for the III_CVT/HOR/ III_Y-axis/FK, where we can presume their mutual connection.

## Discussion

Regarding the Y axis to Frankfurt horizontal angle (Y-axis/FK), a parameter that indicates the mandibular growth direction, significant differences between the patients from groups I-III and II-III can be observed, but no significant differences appear between I-II groups (Fig. 2). These differences can be explained by the fact that generally, patients who have a class II skeletal pattern tend to have a more backward and clockwise rotation of the mandible, while class III patients tend to have a more forward and anticlockwise rotation of the mandible.

The perpendicular distance from H to the mandibular plane (H-ML) is on average 15.722 mm for the class I patients, 13.813 mm for the class II and 16.546 mm for class III patients. There were no statistically significant differences identified, but it seems that the average distance is longer in class III patients than in class II patients which can be explained by the more anterior positioning tendency of the mandible in class III patients. The same situation was observed in the case of the angle between Hyoidale and the mandibular plane (H/ML), which indicates the relation of the hyoid bone to the mandibular plane in the vertical plane. The angle between the mandibular plane and the hyoid bone is 20.333 on average for the class I skeletal patients, 21.00 for the class II and 18.818 for class III patients. As can be seen it is on average higher in class II patients and lower in class III patients which can indicate a higher position tendency of the hyoid bone in class III patients, but no significant statistically changes were identified between the 3 groups. This agrees with Amayeri et al., who did not find significant difference in the angular and linear measurements of the hyoid bone in relation to the mandible in the vertical plane^17^.

The distance from H to Rgn (H-Rgn) is on average 38.889 mm for class I, 29.688 mm for class II and 41.909 mm for class III patients, showing that the distance from H to Rgn is closer for class II patients in relation with class I and further for class III patients compared with class I. This parameter indicates that there is a close connection between the hyoid bone and the positioning of the mandible in the sagittal plane in patients with different skeletal patterns. There are statistically significant differences between all skeletal classes, which can be justified by the more distal positioning of the mandible in class II patients and by the more anterior positioning of the mandible in class III patients. Also, this agrees with Amayeri et al. and Pereira Coelho et all., who found a larger distance in the position of the hyoid bone to Retrognathion in the sagittal plane for class III patients^17,18^.

The distance from H to C3 (H-C3) is on average 33.778 mm in class I, 28.688 mm in class II and 31.546 mm in class III individuals. There were statistically significant differences observed between I and II classes, but no statistical differences between pairs: I-III and II-III. However, a larger distance can be observed in class III patients than in class II patients, but which is nevertheless not statistically significant. These findings disagree with Amayeri et al. who noticed a larger distance alteration between the hyoid bone and the third cervical vertebra in Class III cases than in Class I and Class II cases, showing that there is a close link between hyoid bone-mandible- and the third cervical vertebra, which means that there is also a close connection between dental malocclusion and body posture^17^.

Cervical posture in horizontal plane differs significantly among class II and class III skeletal patients in term of inclination of the upper cervical column to the true horizontal (OPT/HOR). Values reported were: 92.889 in class I, 87.375 in class II and 95.091 in class III. This higher angle in class III patients represents the tendency of this group of patients to a more backward inclination of the cervical column while class II patients tend to have a more forward position of the column.

A significant difference can also be observed between indicators of the middle cervical segment (CVT/HOR) between the pairs I-II and II-III, but between group I-III there were no statistically significant differences. These findings indicate a mildly straighter inclination of cervical vertebra in class III and a more inclined position in class II patients. This agrees with data reported by Hedayati et al., who observed a mildly straighter inclination of the cervical vertebrae in the case of the class III patients^19^.

The average values of skeletal classes regarding the OPT/SN parameters are: 98.889 for class I, 103.500 for class II and 97.273 for class III. There are statistically significant differences in OPT/ SN, between I-II classes and II-III classes, but no differences between I-III classes. The analysis shows that the angles are wider in class II patients compared to class III patients, which means that class II patients may have a more backward head position in relation to the 2nd and 4th cervical vertebra, while class III patients have a more forward head position in relation to the 2nd and 4th cervical vertebra.

CVT/SN has the following values: 101.944 for class I, 104.500 for class II and 99.364 for class III. Significant differences can be observed between II-III classes. Also, these results highlight a more backward position of the anterior cranial base in class II patients and a more forward position of the anterior cranial base in class III patients. Hedayati et al. also reported a more forward position based on the values given by the CVT/SN angle in class III patients. Previous studies have shown that individuals with a retrognathic facial profile (class II skeletal pattern) and an obtuse cranial base angle tends to keep their heads more extended with their chins somewhat protruding, while prognathic facial profiles (class III skeletal pattern) who have a more acute cranial base angle hold their chins inclined and more inferiorly^19,20^.

The angles OPT/ML and CVT/ML have the same tendency, noticing statistically significant differences only between class I patients and class II patients. The OPT/ML angle is lower in class II patients compared to class I patients, which can be justified by a more posterior and backward positioning of the mandible in class II patients than in class I patients. However, there are some differences in the values of the OPT/ML and CVT/ML angles between class II and class III patients, but these are not statistically significant. Class III patients seem to have a higher OPT/ML and CVT/ML angles than class II patients which can be explained by a more forward positioning of the mandible. These values for the angle of the mandibular plane in relation to the cervical column could be explained by the fact that mandibular growth is different among individuals and the mandibular rest position, besides cervical and head posture is also dependent on several factors such as occlusal interferences, temporomandibular dysfunction, psychosocial stress, diurnal variation, and nasal obstruction^21–24^. Also, the counterclockwise rotation of the mandible which is more prevalent in class II patients and the anticlockwise rotation of the mandible, which is more prevalent in class III patients, plays a significant role in determining these angles.

Regarding the linear distance between the anterior cranial base and the hyoid bone (H-SN) there were statistically differences between classes II and III. The values are: 93.611 for class I, 94.00 for class II and 104.546 for class III. The analysis shows that there is a greater H-SN distance in class II patients compared to class I and even greater distance in class III patients compared to class I patients. This means that the hyoid bone position is lower in the vertical plane in relation to the anterior cranial base in class III cases than in class II and I case. This agrees with Amayeri et al.

H/SN angle analysis indicated a significant difference between class II and class III patients, showing that class III skeletal individuals have a more anterior position of the hyoid bone in relation to the anterior cranial base compared to class II individuals. The H/SN angle was 92.722 on average for class I patient, 97.188 in class II patients and 92.727 in class III patients. This agreed with Battagel et al. and Wu et al. who documented a more posterior position of the hyoid bone in Class II skeletal malocclusion subjects and with Adamidis and Spyropoulos, who reported a more anterior position of the Hyoid bone in class III patients^25–27^. This can be attributed to the muscular attachment of the hyoid bone and the mandible. The hyoid follows the mandibular movements in the sagittal plane, so it moves backwards in class II individuals and forward in class III patients.

Natural head posture is the upright position of the head of an individual while the head is in a balanced by the post-cervical, suprahyoid and infrahyoid muscle group^28^. It is dependent on the respiratory function, visual function, and the masticatory muscles. Our cephalometric findings revealed some relationships between the skeletal pattern of a patient, the hyoid bone, head posture and the cervical vertebras. Respectively, we demonstrated that class II patients tend to have a more accentuated cervical kyphosis, with their head position inclined more backward, while class III patients have a more accentuated cervical lordosis with their head position inclined more forward. These findings could explain the relationship between the cervical column posture, head posture and orthodontic malocclusions. The backward positioning of the head in class II patients, may be because of the patient's attempt to compensate the exaggerated kyphosis of the cervical spine resulting in a more distally positioning of the mandible, while in class III patients the more forward positioning of the head, could be the result of the patient’s attempt to compensate for the exaggerated cervical lordosis resulting in a more anteriorly positioning of the mandible.

Regarding the position of the hyoid bone between patients with different vertical growth patterns respectively normodivergent, hyperdivergent and hypodivergent (Fig. 3) according to the FMA angle, our research has revealed some significant differences in some of the parameters evaluated. Vertical position of the hyoid bone in individuals with hyperdivergent growth pattern was slightly lower compared to hypodivergent and normodivergent patients. H-SN value was 93.95 for normodivergent patients, 104.78 for hyperdivergent patients and 89.40 for hypodivergent patients. There were statistically significant differences found in between normodivergent patients and hyperdivergent patients but also between hyperdivergent patients and hypodivergent patients. This agrees with Bhullar et al. who also found that the vertical position of the hyoid bone is slightly uppward in hypodivergent growth pattern patients compared to hyperdivergent patients^29^.

The anteroposterior position of the hyoid in relation to the anterior cranial base identified by the parameter H/SN did not differ among the 3 groups. The values registered were 92.62 in normodivergent, 95.64 in hyperdivergents and 95.30 in hypodivergent individuals.

Hyoid bone position in relation to the mandibular symphysis differ among individuals with different vertical growth patterns. The values registered for the H-Rgn parameter are 37.16 for normodivergent patients, 35.42 for hyperdivergents and 40.1 for hypodivergent patients noticing a more anteriorly displacement of the hyoid bone in hypodivergent individuals. This coincides with Pae et al. ‘s findings^30^.

## Conclusions

Consequently, the most important findings of our paper are:

- Hyoid bone position in relation to retrognathion is closer in class II patients than in class III patients.
- Cervical position differs significantly between class II and class III patients in terms of cervical spine inclination.
- Class III patients have a cervical lordosis tendency, while in class II patients a cervical kyphosis tendency can be observed.
- Class II patients have a more backward inclination of the head, this having a detrimental effect at the occlusal level, through a more posterior positioning of the mandible.
- In class III patients there is an opposite effect. The position of the head being more down and forward most likely as a way of compensating the body for tilting the cervical spine further backwards resulting in a more anterior position of the mandible.

These findings indicate a close relationship between, hyoid bone positioning, cervical and head posture. Therefore, affected patients may compensate for pathological changes at the skeletal level through postural adjustments of the head and neck region. To further evaluate possible applications for orthodontic diagnosis and treatment, more studies should be carried out in this field and to evaluate their possible connections with dental malocclusions.

## Data Availability

All data analyzed during this study are included in this published article.
